# Recurrent multiple amoebic liver abscesses

**DOI:** 10.1186/1471-2334-13-S1-P96

**Published:** 2013-12-16

**Authors:** Adina Ilie, Ramona Popescu, Gabriel Colțan

**Affiliations:** 1 National Institute for Infectious Diseases “Prof. Dr. Matei Balş”, Bucharest, Romania

## Background

We present the management of recurrent liver microabscesses after stopping antibiotic therapy in a patient with no significant pathology associated.

## Case report

A female patient was admitted to our ward for fever 3 days after stopping therapy with ertapenem and metronidazole from a previous hospitalization.

From the medical history we mention that the patient had had two other episodes of liver microabscesses which responded well and promptly to antibiotic regimens that had in common metronidazole. The patient was screened for HIV, HTLV, tuberculosis, endocarditis, hydatid cyst, cancer – results came back negative.

The patient had no context of amoebic liver abscess: Romania is not an endemic area, the patient had not travelled across the border, screening for amoebic coproantigen was negative; all we had to go on was the fact that fever and liver abscesses disappeared under metronidazole treatment alone.

After diagnosis was confirmed, for complete treatment we had to use drugs active also against the intestinal cyst form of *Entamoeba histolytica*, difficult given their limited availability in Romania. After the full course of treatment, the patient has fully recovered.

## Conclusion

When you are confronted with invasive disease caused by *E histolytica* it is mandatory to treat the cystic form.

